# Comparative Genomic Analysis of *Lactiplantibacillus plantarum* Isolated from Different Niches

**DOI:** 10.3390/genes12020241

**Published:** 2021-02-08

**Authors:** Bingyong Mao, Ruimin Yin, Xiaoshu Li, Shumao Cui, Hao Zhang, Jianxin Zhao, Wei Chen

**Affiliations:** 1 State Key Laboratory of Food Science and Technology, Jiangnan University, Wuxi 214122, China; maobingyong@jiangnan.edu.cn (B.M.); yinruiminyeah@163.com (R.Y.); sharonli1994@foxmail.com (X.L.); cuishumao@jiangnan.edu.cn (S.C.); zhanghao61@jiangnan.edu.cn (H.Z.); chenwei66@jiangnan.edu.cn (W.C.); 2 School of Food Science and Technology, Jiangnan University, Wuxi 214122, China; 3 National Engineering Research Center for Functional Food, Jiangnan University, Wuxi 214122, China

**Keywords:** *Lactiplantibacillus plantarum*, comparative genomics, diversity, carbohydrate metabolism

## Abstract

*Lactiplantibacillus plantarum* can adapt to a variety of niches and is widely distributed in many sources. We used comparative genomics to explore the differences in the genome and in the physiological characteristics of *L. plantarum* isolated from pickles, fermented sauce, and human feces. The relationships between genotypes and phenotypes were analyzed to address the effects of isolation source on the genetic variation of *L. plantarum*. The comparative genomic results indicate that the numbers of unique genes in the different strains were niche-dependent. *L. plantarum* isolated from fecal sources generally had more strain-specific genes than *L. plantarum* isolated from pickles. The phylogenetic tree and average nucleotide identity (ANI) results indicate that *L. plantarum* in pickles and fermented sauce clustered independently, whereas the fecal *L. plantarum* was distributed more uniformly in the phylogenetic tree. The pan-genome curve indicated that the *L. plantarum* exhibited high genomic diversity. Based on the analysis of the carbohydrate active enzyme and carbohydrate-use abilities, we found that *L. plantarum* strains isolated from different sources exhibited different expression of the Glycoside Hydrolases (GH) and Glycosyl Transferases (GT) families and that the expression patterns of carbohydrate active enzymes were consistent with the evolution relationships of the strains. *L. plantarum* strains exhibited niche-specific characteristicsand the results provided better understating on genetics of this species.

## 1. Introduction

Lactic acid bacteria (LAB) are microorganisms commonly found in the gut of humans and animals [1]. The major LAB in the intestinal tract of humans are streptococci and enterococci, which are usually regarded as pathogens. Other genera are very minor components (<0.01% of the total bacterial population), such as *Ligilactobacillus*, *Lactobacillus*, and *Limosilactobacillus*. However, *Lactobacillus* and their fermented products in the gut can affect multiple physiological aspects, including the composition of the intestinal microbiota, immune responses, tumorigenesis, the serum cholesterol level, and blood pressure [2].

*L. plantarum* is an LAB that can be found in the gastrointestinal tracts of human and animals, meat, fish, fermented vegetables, and dairy products [3,4,5]. Since the first genome-wide sequencing of *Lactococcus lactis* subsp. *lactis* IL1403 [6], a series of whole-genome sequencing of LAB has been launched worldwide [7,8,9]. With the rapid development of sequencing techniques, the genomes of multiple *L. plantarum* strains have been sequenced [7]. This advancement has provided a better understanding of the relationships among genotypes and functions and the directions of strain evolution [8,9]. Comparative genomics allows horizontal comparison of the genomes of multiple bacterial strains so inter-strain differences can be investigated more efficiently and conveniently [10] in order to enable better functional analysis at the molecular level [11].

Several studies have depicted the genetic diversity of *L. plantarum* strains using different phenotypic and genotypic approaches, such as amplified fragment length polymorphism (AFLP) [12], random amplified polymorphic DNA (RAPD) [13], multilocus sequence typing (MLST) [14], and microarray-based comparative genome hybridization (CGH) [15,16]. These studies revealed that various *L. plantarum* strains generally exhibited high conservation for genes involved in protein and lipid synthesis or degradation and high diversity for genes involved in sugar transport and catabolism.

A previous comparative genomics study on 54 *L. plantarum* strains revealed that *L. plantarum* isolated from various niches exhibited high genetic diversity and distinct phylogenetic patterns [10]. However, a genomic characterization of 108 *L. plantarum* strains using pan-genome analysis revealed no direct connections between genomic traits and the niche [17]. Moreover, the associations between genotypes and phenotypes have not been fully considered and investigated [18].

In this study, we studied the genomics of 133 *L. plantarum* strains isolated from various niches. We analyzed their core genes, pan genes, and phylogenetics (on the basis of homologous genes) and explored the functional genomics from a comparative genomics perspective. Furthermore, to establish an efficient method to rapidly select strains with application potential on the basis of their genetic features, we explored the relationships between genotypes and carbohydrate use capacity.

## 2. Materials and Methods

### 2.1. Chemicals and Reagents

Cellobiose, sorbose, xylose, mannose, glucuronic acid, ribose, trehalose, and fucose were purchased from Sinopharm Chemical Reagent Co., Ltd. (Shanghai, China); arabinose and raffinose were purchased from Sigma-Aldrich (Shanghai) Trading Co., Ltd. (Shanghai, China); and fructooligosaccharides were purchased from Bao Lingbao Biotechnology Co., Ltd. (Dezhou, Shandong Province, China).

### 2.2. L. plantarum Strains

In this study, 324 samples were collected for isolation of *L. plantarum*, including 126 pickles, 32 fermented sauces, and 166 human feces. The samples were serially diluted, plated on solid MRS medium, and cultured at 37 °C for 24 h. The colonies with the characteristics of lactic acid bacteria were picked up, purified, and identified by 16S rRNA sequencing. If multiple strains of *L. plantarum* are isolated and identified in the same sample, only one strain is selected for the genomic analysis. Thus, a total of 114 strains of *L. plantarum* were obtained in this study, including 32/11/71 strains from pickles/fermented sauce/feces, respectively. The strains were reserved at the Culture Collections of Food Microbiology in Jiangnan University.

Meanwhile, 19 strains of *L. plantarum* with a complete genome were selected from the NCBI database, covering a wide range of niches (Table S1).

### 2.3. Draft Genome Sequencing of L. plantarum

*L. plantarum* strains were inoculated in MRS medium and cultured at 37 °C for 12–16 h, and the bacterial sludge was collected by centrifugation at 8000× *g* for 5 min. Draft genome sequencing was performed by Novogene Co., Ltd. (Beijing, China) using an Illumina HiSeq 2000 sequencing platform.

### 2.4. Genomic Assembly, Prediction, and Functional Annotation

SOAPdenovo software was used for genome assembly and verification of single bases, GapCloser software was used to fill gaps within the genome [19,20], the open reading frame (ORF) was predicted using Glimmer and Genmark software [21], and protein BLAST (BLASTp) from Clusters of Orthologous Groups (COG) Proteome Database was used for ORF homology comparison and functional annotation.

### 2.5. Mapping of Homologous Gene Venn Diagram and Phylogenetic Tree Construction

PGAP software was used to calculate the core genome of *L. plantarum* [22]. Orthologous gene prediction was performed on the genome of each strain using OrthoMCL software [23]. The orthologous gene was then aligned using MAFFT v7.3 software [24], and the Venn diagram was generated using Perl script. Phylogenetic trees were constructed on the basis of the 1620 core genes using PHYLIP v3.6 [25].

### 2.6. Average Nucleotide Identity (ANI) Analysis

PYANI was used to calculate the orthologous average nucleotide identity of the genome. PYANI (https://github.com/widdowquinn/pyani) is a Python3 module. We clustered and visualized the obtained matrix using R package heatmap software. ANI is the average value based on the comparisons of all orthologous protein-encoding genes of the pairwise genomes and is a classic method for judging whether strains belong to the same species [26]. Strains with an ANI value of over 95% are generally considered to be of the same species [27].

### 2.7. Carbohydrate-Active Enzyme Analysis

Carbohydrate-active enzyme (CAZy) in *L. plantarum* was annotated with HMMER v3.0 [28]. The target protein sequence was aligned with hmmscan-parser.sh from the dbCAN prediction platform and the best hit was reserved [29]. The genome was considered to contain the corresponding CAZys when the CAZys analysis was consistent with the annotation results of the dbCAN prediction platform. The detailed carbohydrate-active enzyme family information was obtained on the CAZyme (http://www.cazy.org/) [30]. The database mainly included glycoside hydrolase (GHs), glycosyltransferases (GTs), carbohydrate esterases (CEs), carbohydrate-binding enzymes (CBM), auxiliary active enzymes (AAs), and polysaccharide lyases (PLs).

### 2.8. Determination of the Carbohydrate Utilization Capacity of L. plantarum

A series of MRS media were prepared using different carbohydrates as the sole carbon source (10 gL^−1^). The pH was adjusted to 7.0, and bromocresol purple was added to the medium as an acid production indicator (0.75%, *w/v*). A medium with glucose and a carbohydrate-free medium were used as the positive and negative controls, respectively. The sterilized medium was dispensed into sterile 96-well plates (200 uL for each well). The bacterial strains were resuspended in PBS and cultured in the medium (1%) at 37 °C for 12–24 h. The use of carbohydrate by the bacteria was indicated by the color change of the medium (from violet to yellow).

## 3. Results

### 3.1. Genome Characteristics of 133 L. plantarum Strains

The detailed information of the 114 *L. plantarum* strains is shown in Table S1. In total, 11/32/71 strains were isolated from fermented sauce/pickles/feces, respectively. While the 19 reference strains were isolated from various sources, including sauerkraut, baby droppings, brewing, saliva, pickles, dairy products, fermented sausage, raw milk, *Drosophila*, silage, fermented bean paste, feces, fermented tamarind pulp, and cheese. The basic genomic characteristics of *L. plantarum* strains are shown in Table 1. We found that the genomic size of the 114 strains ranged from 2.94 to 3.90 Mb, and the number of genes ranged from 2767 to 3650. The genomic size and gene number of the strains isolated from fermented sources exhibited a narrower range than those isolated from feces. The 19 reference strains had similar genomic size and number of genes to the 114 strains, and specific strains had a larger number of genes. The GC content is an important indicator of bacterial classification and is associated with many genetic characteristics [31]. We found that the GC content of our *L. plantarum* strains ranged from 44.08% to 46.55%, in which seven strains had GC contents over 46% (Table S1). The GC content of the 19 reference strains ranged from 44.08% to 44.90%, which was less than that of the 114 strains.

**Figure 1:**
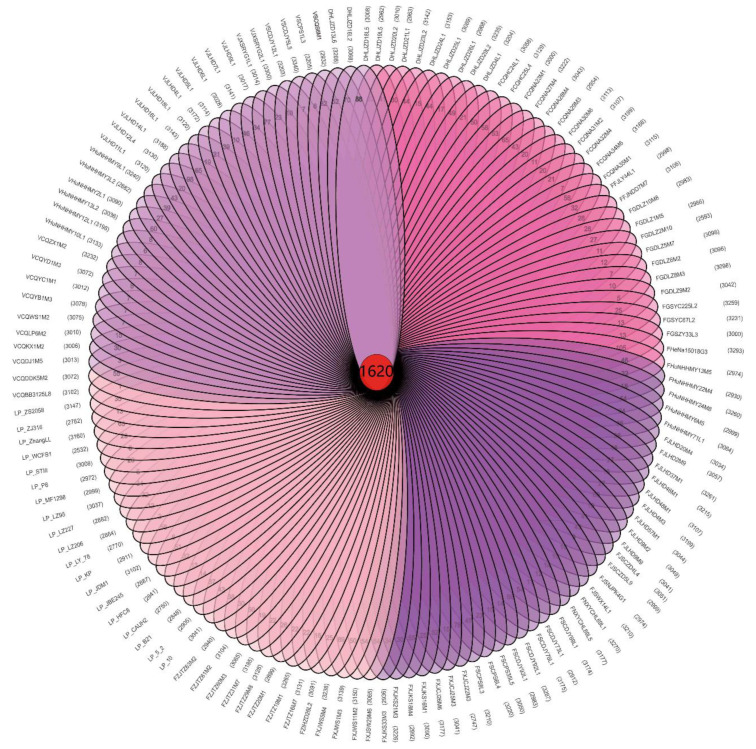
The 1620 core genes and unique genes among 133 *L. plantarum* strains.

The genes of the bacterial strains can be divided into core genes, non-essential genes, and unique genes [32]. We identified 1620 core genes in the 133 *L. plantarum* strains (Figure 1), which was consistent with the previously reported *L. plantarum* core gene value of 1709 [17]. *L. plantarum* had more core genes than other LAB [24,33], which may explain their high adaptability to diverse niches.

### 3.2. Analysis of the Phylogenetic Tree with Regard to Homologous Genes

**Figure 2:**
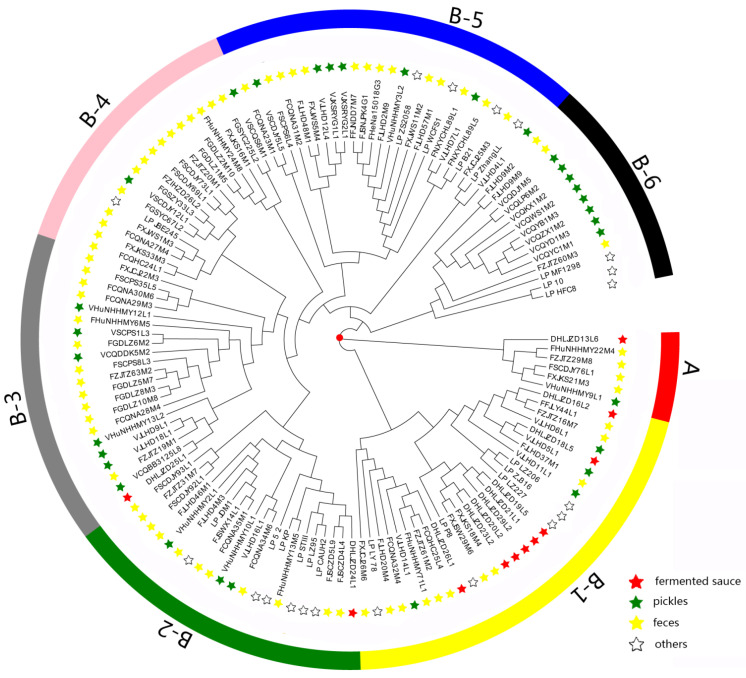
Phylogenetic tree based on sequence similarities among all the 1620 core genes. “Others” stands for the sources of isolation of the 19 reference strains, including sauerkraut, baby droppings, brewing, saliva, pickles, dairy products, fermented sausage, raw milk, *Drosophila*, silage, fermented bean paste, feces, fermented tamarind pulp, and cheese.

Bacterial adaptation to the environment depends not only on the presence or absence of specific genes, but also on the accumulation of specific allelic variations of conserved genes [34]. We conducted phylogenetic analysis of the 133 *L. plantarum* strains to study the role of genetic variation and to reveal their evolutionary relationships (Figure 2).

As shown in Figure 2, the 133 strains fell into two clusters (cluster A and B). Cluster A contained six strains, namely, VHuNHHMY9L1, FXJKS21M3, FSCDJY76L1, FZJTZ29M8, FHuNHHMY22M4, and DHLJZD13L6. We found that the clustering was not affected by various niches in cluster A, possibly due to the small number of strains within the cluster.

Cluster B contained 127 strains and could be subdivided into B-1, B-2, B-3, B-4, B-5, and B-6 clusters. We found that some strains isolated from pickles and fermented sauce were clearly distinguished in the phylogenetic tree. Eight strains isolated from fermented sauce accumulated in the B-1 cluster, five of which aggregated in the same branch under the B-1 cluster. More strains isolated from pickles accumulated in the B-6 cluster than in the B-1 cluster. Interestingly, fecal *L. plantarum* strains exhibited no specific distribution patterns.

The genomic evolution of *L. plantarum* within the same niche was also affected by other factors. For the strains isolated from pickles, eight strains clustered in cluster B-6 and were distinct from other isolates. This may be related to the recycling time and salinity of old brine of different pickles (Table S1), which can affect the composition of microbiota in pickles [35] and may explain the genomic differences in *L. plantarum* strains.

### 3.3. Average Nucleotide Identity (ANI) Analysis

**Figure 3:**
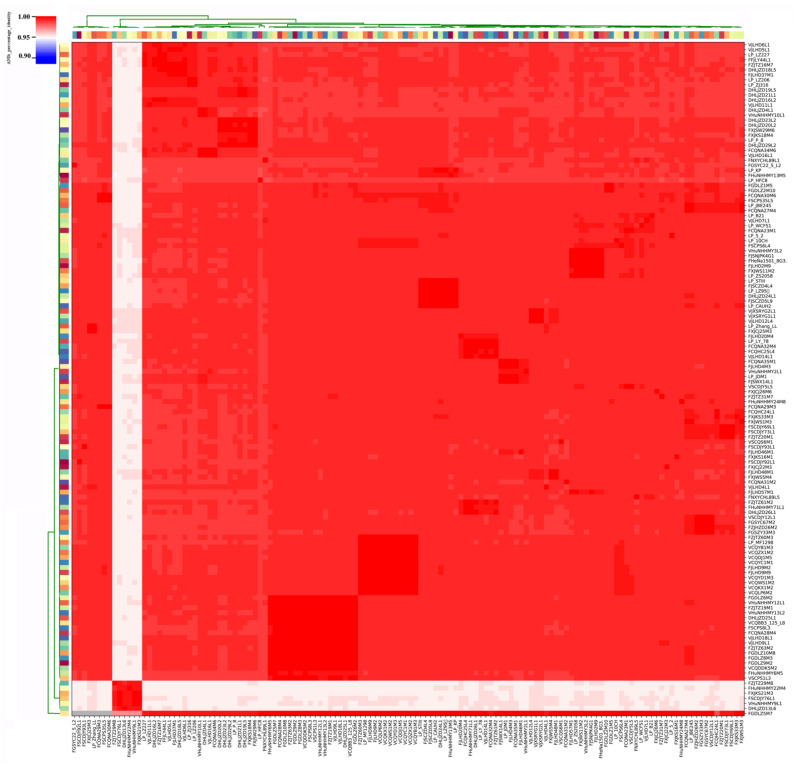
Average nucleotide identity (ANI) identity value for 133 *L. plantarum* strains. The color coding for the strains on the *x*-axis and *y*-axis was used to differentiate the strains.

We found that the ANI values of *L. plantarum* strains isolated from pickles exceeded 99% (Figure 3). Specifically, the ANI values for the eight strains isolated from Chongqing pickles were 99.23%, and the ANI values of *L. plantarum* strains isolated from fermented sauce in Heilongjiang were above 99.23%. However, the ANI values of strains isolated from pickles and fermented sauce were between 98.85% and 99.02%.

Our ANI analysis revealed that six *L. plantarum* strains, namely, FZJTZ29M8, FHuNHHMY22M4, FXJKS21M3, FSCDJY76L1, VHuNHHMY9L1, and DHLJZD13L6, significantly differed from the other 127 strains. The maximum ANI value across these two groups was 95.23%, whereas the ANI values among the six strains were between 99.23% and 99.29%.

The ANI threshold for differentiating strains within the same species was updated to 96% [36] in 2018 (strains with an ANI value greater than 96% are considered the same species). Due to the small difference between our inter-cluster ANI (95.23%) and the accepted threshold, we speculated that these six strains were of a separate species or subspecies in the *L. plantarum* group.

### 3.4. Analysis of Carbohydrate-Active Enzymes

**Figure 4:**
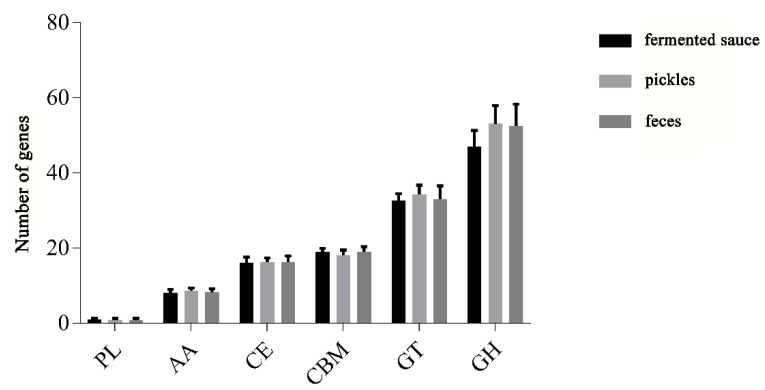
Statistics for carbohydrate-active enzyme (CAZy) family of *L. plantarum* isolated from different niches.

In viewing the annotation results of carbohydrate-active enzymes (CAZy), we found that the 114 *L. plantarum* strains contained all six major families of glycoside hydrolase (GH), glycosyl transferase (GT), carbohydrate esterase (CE), carbohydrate-binding enzyme (CBM), auxiliary active enzyme (AA), and polysaccharide lyase (PL). We found that the most abundant CAZy genes in the *L. plantarum* strains belonged to the GH family, followed by the GT, CBM, CE, AA, and PL families (Figure 4). We observed no significant differences in the number of genes in the families of PL, AA, CE, and CBM for *L. plantarum* strains isolated from different niches.

**Figure 5:**
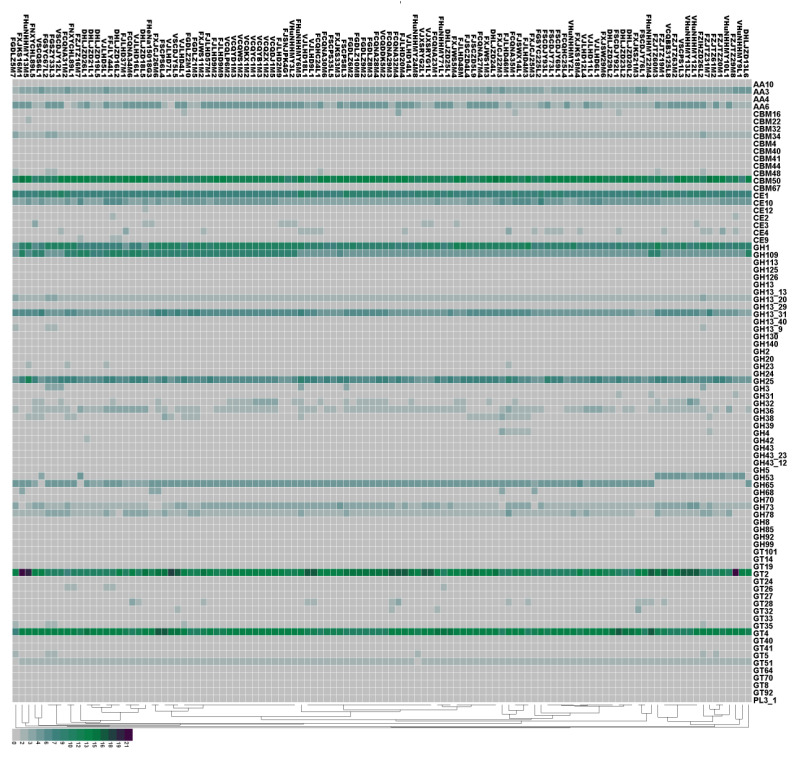
Prediction of carbohydrate-active enzymes (CAZy) in 114 *L. plantarum* strains by protein blast. The isolation sources can be differentiated according to the first letter of the strain names. “F” stands for feces, “V” stands for pickles, and “D” stands for fermented sauce.

To further clarify the differences in CAZy for *L. plantarum* strains isolated from different niches, we next characterized the genes of CAZy using heat maps (Figure 5). Our results indicate that all the strains contained the genes encoding for the AA3 family proteins. The AA3 enzymes belong to the glucose–methanol–choline (GMC) oxidoreductase family and can be divided into four subfamilies: AA3_1 (mostly cellobiose dehydrogenases), AA3_2 (including both aryl alcohol oxidase and glucose 1-oxidase), AA3_3 (alcohol oxidase), and AA3_4 (pyranose 2-oxidase). CBM50 was present in all strains, and was found attached to various enzymes from families GH18, GH19, GH23, GH24, GH25, and GH73, i.e., enzymes cleaving either chitin or peptidoglycan. The GH family were the most abundant enzymes in the CAZy database, which is required for the degradation of glycosidic linkages between carbohydrates [37]. We found that the GH family members encoded in all strains were GH1, GH109, GH13_31, and GH25. The GT family mainly encodes for glycosyltransferases. We identified GT2 (required for cellulose synthesis) and GT4 (required for sucrose synthesis) in all strains.

Our results reveal that *L. plantarum* strains isolated from different niches encoded different CAZy enzymes. Specifically, nine strains isolated from feces in Zhejiang did not contain the GH65 family (required for rhamnose use). Moreover, fecal *L. plantarum* strains had fewer GH36 genes than those isolated from pickles and fermented sauce. The increased number of GH36 genes may help *L. plantarum* strains isolated from pickles to use raffinose, which is a carbohydrate commonly found in plants [38]. The GH43 and GH68 families were found only in the strains isolated from feces, and the GT27 family was found only in the strains isolated from pickles. The GH42 family, which includes β-galactosidase and α-L-arabinosidase, was only found in the strain DHLJZD26L1.

### 3.5. Analysis of Carbohydrate Use Ability

**Figure 6:**
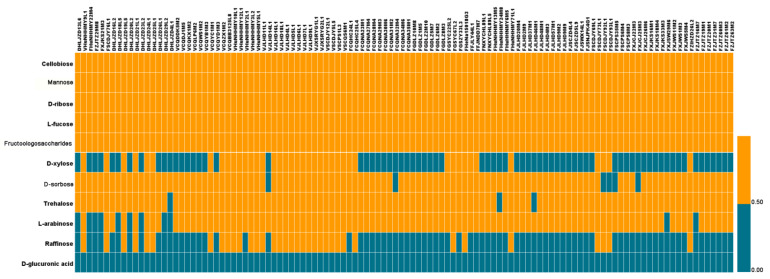
Use of 11 carbohydrates by 114 *L. plantarum* strains.

We further explored the relationships between carbohydrate use-related genes and carbohydrate use capabilities. We analyzed the ability of different *L. plantarum* strains in using 11 carbohydrates (see the Materials and Methods section), including cellobiose, sorbose, xylose, mannose, glucuronic acid, ribose, trehalose, fucose, arabinose, raffinose, and fructooligosaccharides (FOS) (Figure 6). Our results indicate that all *L. plantarum* strains were able to use cellobiose, mannose, ribose, and fucose, which was consistent with the results in Bergey’s Manual of Systematic Bacteriology. Consistent with a previous report [8], we found that all *L. plantarum* strains used FOS. Finally, none of the 114 strains were able to use glucuronic acid, and only 31.25% of the strains could use raffinose.

These findings indicate that the *L. plantarum* strains isolated from different niches showed various abilities to use specific carbohydrates. Although 25% of the *L. plantarum* strains isolated from pickles did not use xylose, 66.67% and 76.39% of *L. plantarum* strains isolated from fermented sauce and feces, respectively, were unable to use xylose. Among the 114 strains, 6 strains, including 5 fecal strains, were unable to use sorbose. We found that three strains did not use trehalose, and 11 strains did not use arabinose, of which five were isolated from fermented sauce in Heilongjiang and six from feces. All strains isolated from pickles were able to use arabinose.

Strains isolated from feces used xylose and raffinose less efficiently than those isolated from pickles and fermented sauce. Thus, our results indicate that the ability of *L. plantarum* to use carbohydrates was strain-specific and, to some extent, niche-specific (dependent on the available carbon sources of that niche).

## 4. Discussion

In this study, comparative genomic analyses were performed for the 133 *L. plantarum* strains isolated from different sources. The phylogenetic tree and ANI analyses indicate that the niche affected the survival and selection, and thus the genomes, of the various *L. plantarum* strains. Indeed, environmental selection pressure promotes genomic modifying processes such as horizontal gene transfer or gene deletion, which improve the adaptability of the strain to a specific niche [39,40,41]. The strains isolated from feces were found in each cluster of the phylogenetic tree (Figure 2), which indicates that the fecal strains showed greater similarity to those isolated from food sources. Siezen et al. studied the sugar-metabolizing capabilities of 185 *L. plantarum* strains isolated from different niches and found that the sugar-metabolizing capability of *L. plantarum* differed according to the food niche, whereas the sugar-metabolizing capability of strains isolated from human feces was not uniform [18]. Consistent with our results from this study, these findings indicated that human fecal *L. plantarum* strains originated from various food sources. We found that a higher proportion of strains isolated from feces diverged later in the phylogenetic tree than those isolated from pickles and fermented sauce, indicating that *L. plantarum* found in the human gut is inoculated via diet.

The use of carbohydrates by *L. plantarum* was not only closely related to the CAZy expressions but also to the expressions of transporters (phosphoglucose transferase (PTS) or ABC transport system) and regulatory proteins. Transporters, regulatory proteins, and related CAZys together constitute an operon that enables sugar metabolism. Wang et al. reported that *L. plantarum* ST-III, WCFS1, and JDM1 contained a large number of phosphoglucose transferase (PTS) systems and displayed broad adaptability to various substrates [42]. In *L. plantarum*, hexose is transported to the cells, phosphorylated by the PTS and permease systems, and fed into the glycolytic pathway and converted to pyruvate [43].

The abilities of the 114 *L. plantarum* strains to use 11 sugars were determined in this study. Our results indicate that all of the strains could use cellobiose, mannose, ribose, fucose, and FOS (Figure 6). According to the CAZy analysis, all of the strains contained 5–12 GH1 families (including β-glucosidase). The reaction catalyzed by β-glucosidase is the most important step in cellobiose degradation. In addition, some strains contained the PTS system, including EIIA, EIIB, EIIC, and HPr. Thus, our findings indicated that all of the *L. plantarum* strains were able to use cellobiose. Mannose-6-phosphoisomerase (EC 5.3.1.8) and the PTS system IIA and IIB (for transporting mannose) are mainly involved in the metabolism of mannose and were found in all the strains. We identified genes involved in ribose metabolism in all strains, such as ABC transporter, purine nucleoside phosphorylase (EC 2.4.2.1), pyrimidine nucleoside phosphorylase (EC 2.4.2.2), and ribose 5-phosphate isomerase A (EC 5.3.1.6). FOS, generally considered as prebiotics, can be used by the intestinal bacteria to produce SCFAs, which have beneficial properties for the host [44]. FOS is made up of a sucrose molecule linked (to its fructose residue) to an extra 1–3 fructose molecules via β-1,2 glycosidic linkages. β-Fructofuranosidase belongs to the GH32 family and is important for the hydrolysis of FOS. In this study, GH32 family was only found in part of the 114 strains, although all the strains metabolized FOS.

Most of the 114 *L. plantarum* strains used trehalose, L-arabinose, and sorbitol, and fewer than half of the strains used xylose and raffinose. None of the strains used glucuronic acid. Trehalose consists of two glucose molecules linked via 1,1 glycosidic bonds, which can be hydrolyzed by α-glucosidase (GH13). In this study, we found that 111 strains used trehalose. L-arabinose is a naturally occurring pentose. In lactobacilli, L-arabinose is transported into the cells via transporters, converted to ribulose (by L-arabinose isomerase—EC 5.3.1.4), and then phosphorylated (by ribulose kinase—EC 2.7.1.16), converted to xylulose phosphate (by L-ribulose-5-phosphate 4-epimerase—EC 5.1.3.4), and finally metabolized. In this study, we observed differences in the use of L-arabinose among the 114 strains. We found that 103 strains used L-arabinose. Genes related to arabinose metabolism were also identified, including L-arabinose isomerase, L-ribulose-5-phosphate 4-epimerase (EC 5.1.3.4), and L-ribulose-5-phosphate 4-epimerase (EC 5.1.3.4). The gene araA (encoding L-arabinose isomerase) was not found in the other 11 strains that did not use L-arabinose. In lactobacilli, the xylose is transported into the cell by the cotransporter Xy1E/Xy1T and the binding protein system Xy1FGH. Intracellular xylose is converted to xylulose by xylose isomerase (encoded by xylA) under the regulation of Xy1R. Xylulose is phosphorylated (to form xylulose-5 phosphate) by xylulose kinase (encoded by xylB), which then enters the pentose phosphate pathway [45]. Our results indicate that only 41 of the 114 strains used xylose. We also identified XylH and the xylulose kinase XylB in these strains. However, xylH was not found in the other 73 strains that did not use xylose. Raffinose is a trisaccharide composed of galactose, glucose, and fructose molecules linked by α-1,6 and α-1,3 glycosidic linkages [46]. The genes for α-galactosidase (EC 3.2.1.22) and β-fructofuranosidase (EC 3.2.1.26), which hydrolyze raffinose, were found in all 114 strains. However, not all strains used raffinose. The gene rafP (encoding for raffinose permease) was only found in the strains capable of using raffinose. In addition, we found that none of the strains use glucuronic acid, probably due to the lack of the GT1 and GT101 families.

## 5. Conclusions

In summary, the comparative genomic results show that the number of strain-specific genes has a certain relationship with the niche in which they are located. The strains from kimchi and fermented sauce were distinctly distinguished, while the strains from feces were mixed in the branches of the phylogenetic tree. The core functions of *L. plantarum* were concentrated on carbohydrate metabolism and amino acid metabolism, which provide molecular support for strains to metabolize multiple sugars. Moreover, we found that the ability of *L. plantarum* strains to use carbohydrates correlated with the carbohydrate metabolism-related genes, including genes encoding glycosidases and transporters. We have previously established that protein BLAST (BLASTp) alignment of the glycosidases and transporters against the protein database of gut bacteria provides effective and reliable predictions on the oligosaccharide-using abilities of gut bacteria [47,48,49]. Further literature mining of the carbohydrate metabolism–related genes would improve the prediction accuracy of this method. Here, we showed that the carbohydrate-using phenotypes of *L. plantarum* strains could be predicted on the basis of certain genetic traits. Finally, we found that a small number of genes did not correspond to the carbohydrate-metabolizing phenotype, probably because the genes were lost in specific niches.

## Figures and Tables

**Table 1:** Basic characteristics of 133 *Lactiplantibacillus plantarum* strains.

Source of Isolation	Number of Strains	Genomic Size (Mb)	Number of Genes	GC Content (%)
Fermented sauce	11	3.14–3.45	3049~3416	45.43–46.33
Pickles	32	3.23–3.50	2931–3464	45.16–46.55
Feces	71	2.94–3.90	2767–3487	45.25–46.29
Others *	19	2.95–3.69	2860–3650	44.08–44.90

* The source of isolation “others” include sauerkraut, baby droppings, brewing, saliva, pickles, dairy products, fermented sausage, raw milk, *Drosophila*, silage, fermented bean paste, feces, fermented tamarind pulp, and cheese. The detailed information is shown in Table S1.

## Data Availability

This study generated sequencing data for 114 *L. plantarum* isolates, and all sequence data have been deposited in the National Center for Biotechnology Information under project no. PRJNA658852; the accession numbers of all isolates are listed in Table S1.

## Supplementary Materials

The following are available online at https://www.mdpi.com/2073-4425/12/2/241/s1: Table S1: Genomic information and sample information of 133 *L. plantarum* strains.
